# Lag Time between Onset of First Symptom and Treatment of Retinoblastoma: An International Collaborative Study of 692 Patients from 10 Countries

**DOI:** 10.3390/cancers13081956

**Published:** 2021-04-19

**Authors:** Swathi Kaliki, Xunda Ji, Yihua Zou, Riffat Rashid, Sadia Sultana, Sadik Taju Sherief, Nathalie Cassoux, Rosdali Y. Diaz Coronado, Juan Luis Garcia Leon, Arturo Manuel Zapata López, Vladimir G. Polyakov, Tatiana L. Ushakova, Soma Rani Roy, Alia Ahmad, Lamis Al Harby, M. Ashwin Reddy, Mandeep S. Sagoo, Jesse L. Berry, Jonathan Kim, Ashley Polski, Nicholas J. Astbury, Covadonga Bascaran, Sharon Blum, Richard Bowman, Matthew J. Burton, Allen Foster, Nir Gomel, Naama Keren-Froim, Shiran Madgar, Andrew W. Stacey, David M. Steinberg, Ashik Mohamed, Marcia Zondervan, Ido Didi Fabian

**Affiliations:** 1 The Operation Eyesight Universal Institute for Eye Cancer, L V Prasad Eye Institute, Hyderabad 500034, India; 2 Department of Ophthalmology, Xinhua Hospital, Shanghai Jiao Tong University School of Medicine, Shanghai 200025, China; jixunda@xinhuamed.com.cn (X.J.); 3147119084@sjtu.edu.cn (Y.Z.); 3 Department of Oculoplasty and Ocular Oncology, Ispahani Islamia Eye Institute and Hospital, Dhaka 1215, Bangladesh; riffat.rashid@islamia.org.bd (R.R.); sadia.sultana@islamia.org.bd (S.S.); 4 Department of Ophthalmology, School of Medicine, Addis Ababa University, Addis Ababa 3614, Ethiopia; sadik.taju@aau.edu.et; 5 Institut Curie, Université de Paris Medicine Paris V Descartes, 75248 Paris, France; nathalie.cassoux@curie.fr; 6 Instituto Nacional de Enfermedades Neoplasicas, Lima 15038, Peru; rosdali.diaz.c@upch.pe (R.Y.D.C.); zapatalopezarturo@gmail.com (A.M.Z.L.); 7 Anglo American Clinic, Lima 15073, Peru; jgarcia@inen.sld.pe; 8 Head and Neck Tumors Department, SRI of Pediatric Oncology and Hematology, N.N. Blokhin National Medical Research Center, Oncology of Russian Federation, 115478 Moscow, Russia; vgp-04@mail.ru (V.G.P.); ushtat07@mail.ru (T.L.U.); 9 Medical Academy of Postgraduate Education, 125445 Moscow, Russia; 10 Chittagong Eye Infirmary & Training Complex, Chittagong 4202, Bangladesh; dr.somaroyceitc@mail.com; 11 The Children’ Hospital & the Institute of Child Health, Lahore 54000, Pakistan; alia.ahmad@stjude.org; 12 The Royal London Hospital, Barts Health NHS Trust, London E1 1BB, UK; lamis.alharby1@nhs.net (L.A.H.); ashwin.reddy4@nhs.net (M.A.R.); m.sagoo@ucl.ac.uk (M.S.S.); 13 Moorfields Eye Hospital NHS Foundation Trust, London EC1V 2PD, UK; 14 UCL Institute of Ophthalmology, London EC1V 2PD, UK; matthew.burton@lshtm.ac.uk; 15 Children’s Hospital Los Angeles & USC Roski Eye Institute, Keck School of Medicine, University of Southern California, Los Angeles, CA 90027, USA; jesse.berry@med.usc.edu (J.L.B.); jonkim@chla.usc.edu (J.K.); ashley.polski@usc.edu (A.P.); 16 International Centre for Eye Health London School of Hygiene & Tropical Medicine, London WC1E 7HT, UK; nick.astbury@lshtm.ac.uk (N.J.A.); covadonga.bascaran@lshtm.ac.uk (C.B.); richard.bowman@lshtm.ac.uk (R.B.); allen.foster@lshtm.ac.uk (A.F.); marcia.zondervan@lshtm.ac.uk (M.Z.); idofabia@post.tau.ac.il (I.D.F.); 17 Goldschleger Eye Institute, Sheba Medical Center, Tel Hashomer, Tel-Aviv University, Tel-Aviv 52621, Israel; sharonblum@tlvmc.gov.il (S.B.); shiran.madgar@sheba.gov.il (S.M.); naama.keren@sheba.health.gov.il (N.K.-F.); 18 Ophthalmology Department, Great Ormond Street Children’s Hospital, London WC1N 3JH, UK; 19 Division of Ophthalmology, Tel Aviv Sourasky Medical Center, Sackler Faculty of Medicine, Tel-Aviv University, Tel-Aviv 39040, Israel; nirgom@tlvmc.gov.il; 20 Department of Ophthalmology, University of Washington, Seattle, WA 98195, USA; awstacey@uw.edu; 21 Department of Statistics and Operations Research, School of Mathematical Sciences, Raymond and Beverly Sackler Faculty of Exact Sciences, Tel Aviv University, Tel Aviv 69978, Israel; dms@tauex.tau.ac.il; 22 Ophthalmic Biophysics, L V Prasad Eye Institute, Hyderabad 500034, India; ashikmohamed@lvpei.org

**Keywords:** eye, tumour, retinoblastoma, lag time, national income level

## Abstract

**Simple Summary:**

The authors aimed to determine the lag time between onset of symptoms and diagnosis of retinoblastoma in countries based on their national-income and analyse its effect on the outcomes. Based on analysis of 692 retinoblastoma patients from 11 treatment centres in 10 countries, there was a statistically significant difference in the lag time between onset of symptoms and diagnosis of retinoblastoma based on country income level. This difference in the lag time between different countries results in varied outcomes across patients. Shorter lag time results in better chances of eye and patient survival.

**Abstract:**

Background: The relationship between lag time and outcomes in retinoblastoma (RB) is unclear. In this study, we aimed to study the effect of lag time between onset of symptoms and diagnosis of retinoblastoma (RB) in countries based on their national-income and analyse its effect on the outcomes. Methods: We performed a prospective study of 692 patients from 11 RB centres in 10 countries from 1 January 2019 to 31 December 2019. Results: The following factors were significantly different among different countries based on national-income level: age at diagnosis of RB (*p* = 0.001), distance from home to nearest primary healthcare centre (*p* = 0.03) and mean lag time between detection of first symptom to visit to RB treatment centre (*p* = 0.0007). After adjusting for country income, increased lag time between onset of symptoms and diagnosis of RB was associated with higher chances of an advanced tumour at presentation (*p* < 0.001), higher chances of high-risk histopathology features (*p* = 0.003), regional lymph node metastasis (*p* < 0.001), systemic metastasis (*p* < 0.001) and death (*p* < 0.001). Conclusions: There is a significant difference in the lag time between onset of signs and symptoms and referral to an RB treatment centre among countries based on national income resulting in significant differences in the presenting features and clinical outcomes.

## 1. Introduction

Retinoblastoma (RB) is the most common intraocular malignancy in children worldwide. The global incidence of RB is estimated at 1 in 15,000 to 20,000 live births [1,2,3,4,5]. The age-standardised incidence rate of RB for children aged 0–5 years in most developed countries is 3–5 cases per million population, while, in developing and underdeveloped countries, the incidence rate is reported to be higher at 6–10 cases per million population [6,7,8,9]. There is a vast difference in the mortality rate between continents, with 70% mortality rate in Africa, 39% in Asia and 3–5% in Northern America and Europe [5]. This difference in the death rates due to RB is mainly attributed to delayed diagnosis. One likely factor contributing to delay in diagnosis is increased lag time between onset of symptoms and treatment of RB. 

The cause of increased lag time between onset of symptoms and treatment of RB could be multifactorial, related to social, cultural, financial, parental and healthcare associated factors [10]. Studies from various countries have highlighted the importance of early diagnosis of RB and the implications of delayed treatment. Studies from Asia have shown that a delayed diagnosis of RB results in an advanced disease at presentation with poorer chances of globe salvage [11,12]; studies from the United Kingdom and India have revealed higher chances of high-risk histopathological features with subsequent need for adjuvant chemotherapy [13,14]; a study from Brazil has shown increased mortality due to increased lag time [15]; a study from Switzerland has shown that reduced lag time is associated with early stage of the disease at presentation [16]; and a study from the Netherlands has shown correlation between early diagnosis and decreased mortality and blindness [17]. Herein, we prospectively studied the lag time between onset of symptoms and diagnosis of RB in 10 countries on five continents from 1 January 2019 to 31 December 2019 and compared the results based on country income levels to assess the causes of increased lag time and its effect on the patient outcomes. 

## 2. Results

**Figure 1:**
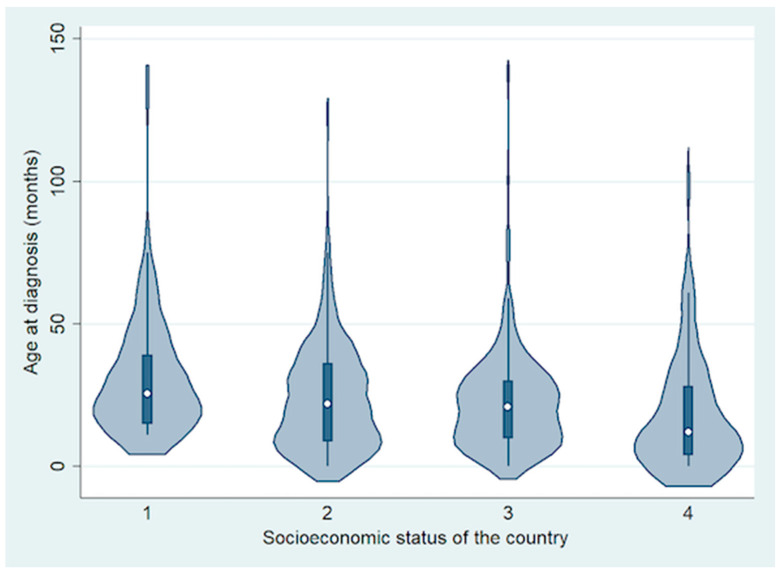
Violin plot showing the difference in age at diagnosis based on socioeconomic status of the country.

In total, 692 patients were included from 10 countries, including 74 (11%) from a low-income country (LIC) (Ethiopia), 294 (42%) from lower middle-income countries (LMIC) (Bangladesh, India and Pakistan), 254 (37%) from upper middle-income countries (UMIC) (China, Peru and Russia) and 70 (10%) from high-income countries (HIC) (France, UK and USA) [18]. Of the 692 patients, 369 (53%) were males and 323 (47%) were females. The mean age at diagnosis of RB was 24 months (median, 21 months; range, <1 to 140 months) (Figure 1). 

**Figure 2:**
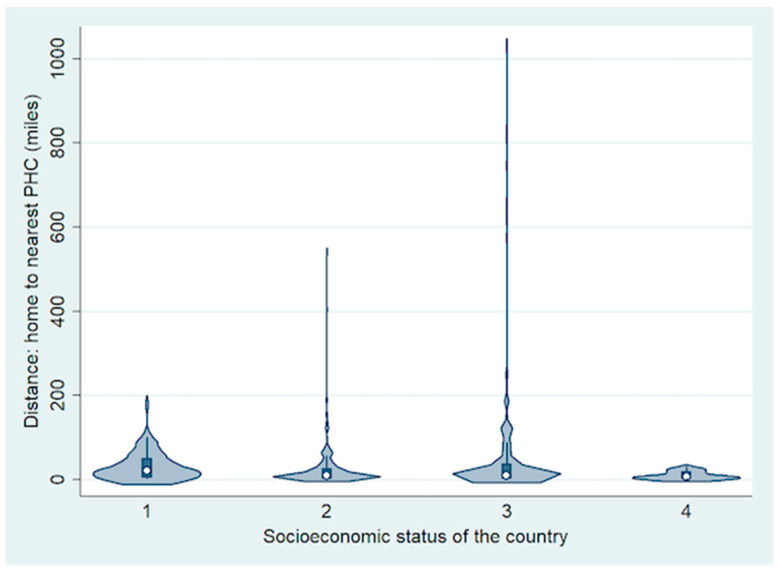
Violin plot showing the difference in distance from home to the nearest primary healthcare centre (PHC) based on socioeconomic status of the country.

The most common symptom of RB was leukocoria (*n* = 508, 73%) followed by strabismus (*n* = 126; 18%). The symptoms were most commonly detected by parents (*n* = 545, 79%) followed by other family members (*n* = 78; 11%). Overall, the mean number of primary healthcare professionals (PHPs) involved before referral to RB treatment centre was 1 (median, 1; range, 0–4), including 2 (1, 1–3) for LIC, 2 (1, 0–4) for LMIC, 1 (1, 0–4) for UMIC and 1 (1, 0–3) for HIC. Of the 692 children, 396 (57%) were diagnosed as RB after the first visit to the primary healthcare professional, 169 (24%) were diagnosed during the second visit to the same or a different primary healthcare professional, 49 (7%) during the third visit and 14 (2%) during the fourth visit, and they were referred to an RB treatment centre for appropriate treatment. The remaining 64 (9%) patients were diagnosed with RB at the RB treatment centre directly. There was family history of RB in three children (one from HIC and two from LMIC), and they were detected to have RB after routine fundus screening. Overall, the mean distance (miles) from home to RB treatment centre was 365 (median, 195; range, 2–9757) (Figure 2). 

**Figure 3:**
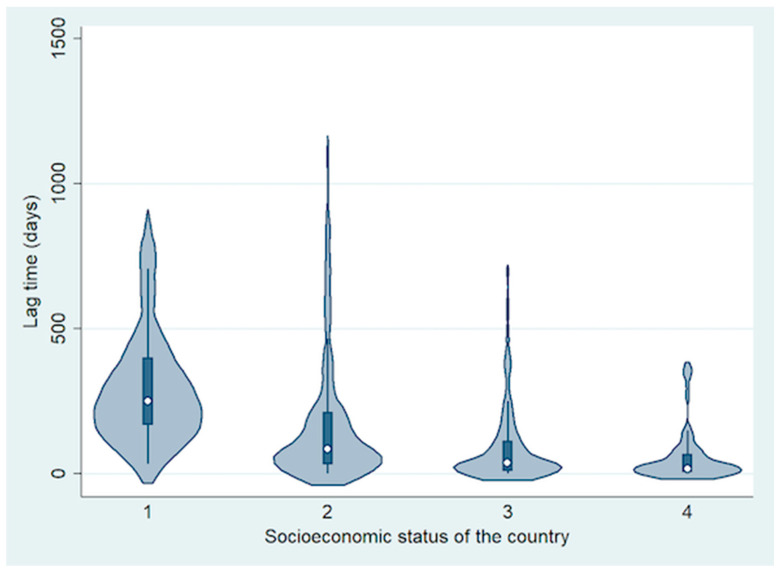
Violin plot showing the difference in lag time from first symptom to treatment based on socioeconomic status of the country.

The mean lag time between detection of first symptom to visit to RB treatment centre was 150 days (median, 69 days; range, 0–1128 days) (Table 1 and Figure 3). Based on analysis by country income level, the mean lag time decreased with increasing national income level. It was 303 days for LIC (median, 251 days; range, 33–846 days), 180 days for LMIC (median, 86 days; range, 0–1128 days), 92 days for UMIC (median, 37 days; range, 1–697 days) and 56 days for HIC (median, 18 days; range, 0–366 days) (difference between groups; *p* = 0.0007). 

The tumour was unilateral at initial presentation in 490 (71%) patients and bilateral in 202 (29%) patients. Of 894 eyes with RB, the tumour was intraocular in 815 (91%) eyes and was associated with extraocular tumour extension in 79 (9%) eyes. Based on AJCC classification [19], the tumour was classified as cT1 in 124 (14%), cT2 in 397 (44%), cT3 in 294 (33%) and cT4 in 79 (9%) eyes. Advanced RB (AJCC cT4) was noted in 20 (20%) eyes from LIC, 36 (9%) from LMIC, 23 (7%) from UMIC and 0 (0%) from HIC (*p* = 0.0004). 

Of the 866 eyes treated for RB, globe salvage was achieved in 513 (59%) eyes during the study period, including 27 (30%) eyes in LIC, 229 (58%) eyes in LMIC, 197 (62%) eyes in UMIC and 58 (68%) eyes in HIC (*p* < 0.0001). Of 353 enucleated eyes, histopathologic high-risk RB features were noted in 179 (51%) eyes, including 40 (63%) eyes in LIC, 75 (46%) eyes in LMIC, 61 (50%) eyes in UMIC and 3 (11%) eyes in HIC. Spread to regional lymph nodes was noted in 20 (3%) patients, systemic metastasis (including central nervous system) in 38 (5%) patients and death occurred in 21 (3%) patients by the conclusion of the study (Table 2). 

On a multivariate analysis, the factors significantly associated with increased lag time between onset of symptoms and diagnosis of RB included lower-national income level (*p* < 0.001), increased number of visits to primary healthcare centres (*p* < 0.001), increased distance from home to RB treatment centre (*p* = 0.02), strabismus as the first symptom of RB (*p* = 0.001) and increasing age at diagnosis (*p* < 0.001). An increase in the level of country income decreased the lag time by 75 days; every 33 miles increase in the distance from home to RB centre increased the lag time by 1 day; every extra visit to a healthcare centre increased the lag time by 36 days; the presence of strabismus as the first symptom of RB increased the lag time by 57 days; and an increase in age of diagnosis by one month increased the lag time by 3 days (Table 3). 

After adjusting for the country’s income, increased lag time between onset of symptoms and diagnosis of RB was associated with higher chances of an advanced T4 tumour at presentation (*p* < 0.001), higher chances of high-risk histopathology features (*p* = 0.003), regional lymph node metastasis (*p* < 0.001), systemic metastasis (*p* < 0.001) and death at time of presentation (*p* < 0.001) (Table 4).

## 3. Discussion

National income level is an important parameter that may be associated with the patient, family and healthcare professional’s education about a disease, accessibility and availability of appropriate care, which may in turn influence the outcomes. There is a significant disparity in the presentation patterns of RB depending on the country’s economic grouping. A study of 4351 RB patients from 153 countries from different national income status revealed that patients from LIC had a larger proportion of patients with signs of advanced disease compared to patients from HIC, patients from LIC and LMIC were older at the time of RB diagnosis, and patients from HIC were more commonly associated with intraocular and earlier stage disease while extraocular disease was more common in children from LIC [20]. Similar findings were noted in the present study. Children in LIC were older compared to those in UMIC and HIC at the time of diagnosis of RB; symptoms of advanced disease such as proptosis or fungating mass was more common in LIC compared to HIC; and advanced tumour (cT4) was more common in LIC compared to LMIC or UMIC or HIC.

The disparity in presentation patterns between different countries can affect the outcomes. There is a significant disparity in the outcomes of RB between LMIC/UMIC and HIC. In a global study of 2085 patients from 18 retinoblastoma centres from 13 countries on six continents, including patients from LMIC, UMIC and HIC, it was noted that the metastasis-related mortality rate was 9–10 times higher in children from UMIC/LMIC compared to HIC. The risk of treatment failure (requiring enucleation or external beam radiotherapy) was two-fold higher in children from UMIC and LMIC compared to those from HIC [21]. Enucleation may not always be due to treatment failure but due to non-availability of other treatment options such as intra-arterial or intra-vitreal chemotherapy in some countries. In our study, which included patients from LIC, LMIC, UMIC and HIC, it was noted that there was an inverse relationship between the risk of metastasis and the need for primary/secondary enucleation and the national income status. Patient survival and globe salvage was better in children from HIC compared to those from LIC at the conclusion of this study. 

Early diagnosis and treatment of RB results in favourable outcomes with better chances of vision, globe and life salvage. Advanced disease presentation and poor patient outcomes are related to increased lag time before initiation of RB treatment. In our study, there was a significant difference in the lag time between first symptom and initiation of RB treatment for LIC vs. LMIC vs. UMIC vs. HIC. The lag time between first symptom and treatment of RB was 5.4 times higher in LIC, 3.2 times higher in LMIC and 1.6 times higher in UMIC when compared to HIC (*p* = 0.0007). On multivariate analysis, the factors influencing increased lag time between onset of symptoms and diagnosis of RB included lower-national income level, increased number of visits to primary healthcare centres, increased distance from home to RB treatment centre, strabismus as the first symptom of RB and increasing age. In a comparative study of RB patients from European countries vs. African countries, it was shown that, despite shorter distance of travel for RB care, African patients presented with more advanced disease at presentation compared to those in Europe [22]. The risk factors for advanced disease included lower-national income level and older age and not the distance of travel for care [22]. In our study, although increased distance from home to RB centre was a significant factor in causing increased lag time, lower-national income level was also significant. Increased number of visits to non-RB centres prior to referral to RB centre indicates probable misdiagnosis at non-RB centres resulting in delayed referral to the appropriate RB centre. These early encounters without a diagnosis being made are a missed opportunity to address the problem in a timelier manner. These findings suggest that it is important to increase awareness about RB among parents or family members and general practitioners, to allow early diagnosis and early referral. 

The most common symptom of RB in HIC was reported to be leukocoria (56–97%), while, in LIC, it was proptosis (65% to 85%) [11]. Strabismus is the second most common symptom of RB in HIC (24%) [23], while it is less common in UMIC, LMIC and LIC [11]. In our study, it was noted that, when strabismus was the first symptom of RB, there was an increased lag time before initiation of RB treatment. Whenever strabismus is noted in a child, red reflex testing or fundus evaluation is mandatory to rule out RB and other causes which could result in amblyopia. In our study, increasing age also resulted in increased lag time for RB treatment. This could be related to atypical symptoms of RB such as decreased vision, enlarged eyeball or eyelid swelling, which are more common in older children compared to younger children [24]. 

Independent of the national income level, ethnicity or socioeconomic status, increased lag time before initiation of RB treatment results in poorer outcomes [10,25]. In our study, when the effect of national income level was adjusted, the consequences of increased lag time between symptoms and diagnosis of RB included advanced tumour at presentation, higher chances of high-risk histopathology features, regional lymph node and systemic metastasis and metastasis-related mortality. Although there was a significant association between increased lag time and high-risk RB in our study, studies from UK [26] and the US [27], have shown that there is no significant association between increased lag time and high-risk histopathology features. This contrasting finding is likely related to the much shorter lag time from first symptom to RB treatment centre in the high-income UK and US groups (median of 31 days in the UK study and 45 days in the US study) compared to this study (median lag time of 69 days, with a median of 251 days and 86 days in LIC and LMIC, respectively). 

Based on the results of our study, it is clear that there is a huge disparity in the lag time between different countries resulting in varied outcomes. An effective first step towards decreasing lag time and thereby improving survival in RB within a country is via targeted awareness campaigns. RB education programs could effectively reduce the extraocular disease from 73% to 35% within two years in Honduras [28] and from 56% to 17% in Brazil [29]. Similarly, in the UK, public awareness campaigns have improved the median lag time from eight weeks in the 1990s to five weeks in the 2010s [14,26].

The limitations of the study include unequal distribution of patients across countries and inadequate follow-up period to derive accurate results on final outcomes since this was a prospective short-term study. It is possible that events such as metastasis or death would have occurred after conclusion of the study. In addition, the patients were grouped based on country-income levels, and there is an expected variation in individual family income within each group, which may cause variation of results within the same group. 

## 4. Materials and Methods

This study was a collaboration of 11 RB treatment centres located in 10 countries from five continents. The study was approved by the London School of Hygiene & Tropical Medicine Institutional Review Board (reference No. 15882). All participating centres received clearance from their respective institutional review board and ethics committee for participating in this international collaborative study. The study adhered to the tenets of Declaration of Helsinki. Informed consent was obtained from all parents/guardians of the children included in this study. It was a one-year prospective study that included all treatment-naïve RB patients who presented to the participating centres from 1 January 2019 to 31 December 2019, and who were treated or offered treatment for RB. All patients who had received prior treatment were excluded from this study.

The countries were classified as low-income country (LIC), lower middle-income countries (LMIC), upper middle-income countries (UMIC) or high-income countries (HIC) based on the United Nations World population prospects (2017 revision) [18]. Using a predesigned form, data were collected prospectively as patients presented to the participating centre. The collated data included the patient’s country of residence, sex, neonatal history, date of first symptom, visit to primary healthcare professionals, examination at presentation to RB treatment centre, distance from home to the RB centre and primary treatment given (see appendix/supplement for a list of the retrieved parameters). Lag time was defined as the duration between the first symptom (as noticed by parents/family members/others) to treatment of RB at the RB treatment centre. For uniform staging, the 8th edition of the American Joint Committee on Cancer (AJCC) clinical Tumor, Node, Metastasis, Heredity (cTNMH and pTNM) system [19] was used. 

### Statistical Analysis

The statistical analysis was performed using R software and STATA v14.2 (StataCorp, College Station, TX, USA). Descriptive measures included mean, median, range and proportion. Data were categorised based on the socioeconomic status of the country; variables were compared by mixed effects model with random intercepts at continent, country and patient levels. Relationships between lag time and other variables were evaluated by multilevel mixed effects linear regression after adjustment for socioeconomic status of the country. A *p*-value of <0.05 was considered statistically significant. A Bonferroni correction was used to account for multiple pairwise comparisons among four categories of country based on income. The resulting Bonferroni adjusted alpha level of 0.017 was used, resulting in a *p*-value of <0.017 being considered statistically significant.

## 5. Conclusions

This is the first international multi-institutional study to compare the difference in the lag time between symptoms and diagnosis of RB between different countries as well as its influence on the outcomes. There is a huge disparity in the lag time between symptoms and diagnosis of RB among different countries depending on the country’s income level. Increased lag time before initiation of RB treatment results in advanced disease at presentation and consequently poorer outcome. While availability and accessibility of healthcare facilities may differ based on national income level, programs focusing on increasing awareness about RB among the care givers and general practitioners would play a crucial role in decreasing the lag time and improving patient outcomes. Routine fundus screening of at-risk patients (i.e., with positive family history of RB) or those presenting with strabismus should be encouraged to ensure early diagnosis of RB and minimise lag time. 

## Figures and Tables

**Table 1:** Demographics and presenting features of 692 retinoblastoma patients from 10 countries.

Feature	All Cases *n* = 692	Low-Income Country (LIC) *n* = 74	Lower Middle-Income Countries (LMIC) *n* = 294	Upper Middle-Income Countries (UMIC) *n* = 254	High Income Countries (HIC) *n* = 70	*p*-Value
Age at diagnosis (months) Mean (median, range)	24 (21, <1 to 140)	31 (26, 11–136)	24 (23, <1 to 125)	22 (21, <1 to 140)	21 (15, <1 to 106)	0.001 ^a^
Sex						
Male	369 (53)	41 (55)	154 (52)	135 (53)	39 (56)	0.94
Female	323 (47)	33 (45)	140 (48)	119 (47)	31 (44)	
Distance from home to nearest primary healthcare centre (miles) Mean (median, range)	34 (10, <1 to 1040)	34 (22, 1–187)	24 (10, <1 to 546)	47 (10, <1 to 1040)	10 (6, <1 to 31)	0.03 ^b^
Distance from home to retinoblastoma centre (miles) Mean (median, range)	365 (195, 2–9757)	244 (227, 2–932)	254 (130, 2–1774)	527 (424, 2–4922)	372 (98, 2–9757)	0.25
First symptom *						
Leukocoria	508 (73)	70 (95)	235 (80)	168 (66)	35 (50)	0.09
Strabismus	126 (18)	5 (7)	53 (18)	52 (20)	16 (23)	0.09
Others **	100 (14)	4 (5)	31 (11)	44 (17)	21 (30)	0.002 ^c^
First symptom noticed by						
Parents	545 (79)	71 (96)	234 (80)	193 (76)	47 (67)	0.01 ^d^
Other family members	78 (11)	1 (1)	41 (14)	34 (13)	2 (3)	0.47
Others	69 (10)	2 (3)	19 (6)	27 (11)	21 (30)	<0.0001 ^e^
Number of visits to primary healthcare centres before referral to RB centre Mean (median, range)	1 (1, 0–4)	2 (1, 1–3)	2 (1, 0–4)	1 (1, 0–4)	1 (1, 0–3)	0.44
Lag time between first symptom and visit to RB centre (days) Mean (median, range)	150 (69, 0–1128)	303 (251, 33–846)	180 (86, 0–1128)	92 (37, 1–697)	56 (18, 0–366)	0.0007 ^f^

RB, retinoblastoma; * Total is >100% since few patients had more than one symptom; ** other symptoms included low vision, red eye, watering, iris heterochromia, proptosis and eyelid swelling. ^a^ LIC vs. UMIC (*p* = 0.008); LIC vs. HIC (*p* = 0.002). ^b^ LMIC vs. UMIC (*p* = 0.008). ^c^ HIC vs. LIC (*p* = 0.001); HIC vs. LMIC (*p* = 0.001). ^d^ LMIC vs. HIC (*p* = 0.007). ^e^ HIC vs. LIC (*p* < 0.001); HIC vs. LMIC (*p* < 0.001); HIC vs. UMIC (*p* < 0.001). ^f^ LIC vs. UMIC (*p* = 0.007); LIC vs. HIC (*p* < 0.001); LMIC vs. HIC (*p* = 0.002).

**Table 2:** Clinical features, treatment and outcomes of 692 retinoblastoma patients from 10 countries.

Feature	All Cases *n* = 894 Eyes in 692 Patients	Low-Income Country (LIC) *n* = 98 Eyes of 74 Patients	Lower Middle-Income Countries (LMIC) *n* = 392 Eyes of 294 Patients	Upper Middle-Income Countries (UMIC) *n* = 319 Eyes of 254 Patients	High Income Countries (HIC) *n* = 85 Eyes of 70 Patients	*p*-Value
Tumour laterality						
Unilateral	490 (71)	50 (68)	196 (67)	189 (74)	55 (79)	0.09
Bilateral	202 (29)	24 (32)	98 (33)	65 (26)	15 (21)	
8th edition AJCC						
Tumour						
T1	124 (14)	20 (20)	43 (11)	41 (13)	20 (24)	0.006 ^b^
T2	397 (44)	33 (34)	144 (37)	175 (55)	45 (53)	0.23
T3	294 (33)	25 (26)	169 (43)	80 (25)	20 (24)	0.53
T4	79 (9)	20 (20)	36 (9)	23 (7)	0 (0)	0.0004 ^c^
Lymph nodes						
N0	672 (97)	60 (81)	289 (98)	253 (99)	70 (100)	<0.0001 ^a^
N1	20 (3)	14 (19)	5 (2)	1 (<1)	0 (0)	
Metastasis						
M0	654 (95)	62 (84)	274 (93)	248 (98)	70 (100)	0.13
M1	38 (5)	12 (16)	20 (7)	6 (2)	0 (0)	
Primary treatment *						
Focal treatment **	132 (15)	20 (20)	35 (9)	55 (17)	22 (26)	0.27
IVC	381 (43)	18 (18)	204 (52)	131 (41)	28 (33)	0.51
IAC	121 (14)	0 (0)	2 (<1)	97 (30)	22 (26)	0.005 ^d^
IviC	27 (3)	0 (0)	1 (<1)	20 (6)	6 (7)	<0.0001 ^e^
Enucleation	279 (31)	51 (52)	135 (34)	68 (21)	25 (29)	0.35
Treatment refusal	28 (3)	7 (7)	9 (2)	3 (<1)	0 (0)	0.03 ^f^
Outcomes at the end of the study						
Globe salvage	513 (59)	27 (30)	229 (58)	197 (62)	58 (68)	<0.0001 ^g^
HRF	179 (51)	40 (63)	75 (46)	61 (50)	3 (11)	0.31
Death	21 (3)	0 (0)	16 (5)	5 (2)	0 (0)	0.26

AJCC, American Joint Committee Classification; EBRT, external beam radiotherapy; * Total is more than 100% since few patients received more than one form of primary treatment; ** argon laser photocoagulation or cryotherapy; IVC, intravenous chemotherapy; IAC, intra-arterial chemotherapy; IviC, intravitreal chemotherapy; HRF, high-risk histopathologic features. ^a^ LIC vs. LMIC (*p* < 0.001); LIC vs. UMIC (*p* < 0.001); LIC vs. HIC (*p* < 0.001). ^b^ HIC vs. LMIC (*p* = 0.003); HIC vs. UMIC (*p* = 0.014). ^c^ LIC vs. LMIC (*p* = 0.011); LIC vs. UMIC (*p* = 0.002); LIC vs. HIC (*p* < 0.001); LMIC vs. HIC (*p* = 0.009). ^d^ LMIC vs. HIC (*p* = 0.001). ^e^ LMIC vs. UMIC (*p* < 0.001); LMIC vs. HIC (*p* < 0.001). ^f^ LIC vs. HIC (*p* = 0.016). ^g^ LIC vs. LMIC (*p* < 0.001); LIC vs. UMIC (*p* < 0.001); LIC vs. HIC (*p* < 0.001).

**Table 3:** Multivariate linear regression analysis of factors affecting lag time between first symptom and treatment of retinoblastoma in 692 patients from 10 countries.

Feature	*p*-Value	Variable Co-Efficient
National income level	<0.001	−74.8 ± 18.3
Number of visits to primary healthcare centres before referral to RB centre	<0.001	35.9 ± 9.9
Distance from home to retinoblastoma centre (miles)	0.02	0.03 ± 0.01
Strabismus as the first symptom of RB	0.001	57.2 ± 17.3
Age at diagnosis (months)	<0.001	2.6 ± 0.4

RB, retinoblastoma.

**Table 4:** Multivariate analysis of marginal linear predictions of factors affected by lag time between first symptom and treatment of retinoblastoma in 692 patients from 10 countries, adjusted for country income level.

Feature	*p*-Value	Variable Co-Efficient
Tumour category based on 8th edition AJCC		
T1 *	0.04	-
T2	0.004	−0.0003 ± 0.00009
T3	0.20	-
T4	<0.001	0.0005 ± 0.00005
Regional lymph node involvement	<0.001	0.0003 ± 0.00001
Metastasis	<0.001	0.0004 ± 0.00005
Primary treatment		
Focal treatment *	0.03	-
IVC	0.54	-
IAC	0.49	-
IviC	0.55	-
Enucleation	0.31	-
Outcomes at the end of the study		
Globe salvage	0.23	-
HRF	0.003	0.0004 ± 0.0001
Death	<0.001	0.0002 ± 0.00004

AJCC, American Joint Committee on cancer; T, tumour; IVC, intravenous chemotherapy; IAC, intra-arterial chemotherapy; IviC, intravitreal chemotherapy; HRF, high-risk histopathology features. * *p*-value for the model was not statistically significant at 0.05.

## Data Availability

The data presented in this study are available on request from the corresponding author. The data are not publicly available due to ethical concerns.
